# Assessment of liver and renal function tests among gasoline exposed gas station workers in Mekelle city, Tigray region, Northern Ethiopia

**DOI:** 10.1371/journal.pone.0239716

**Published:** 2020-10-09

**Authors:** Tsegay Asefaw, Mistre Wolde, Abebe Edao, Aster Tsegaye, Gebre Teklu, Feven Tesfay, Gebreslassie Gebremariam

**Affiliations:** 1 Department of Medical Laboratory Science, College of Health Sciences, Mekelle University, Mekelle, Ethiopia; 2 Department of Medical Laboratory Sciences, College of Health Science, Addis Ababa University, Addis Ababa, Ethiopia; Oregon State University, UNITED STATES

## Abstract

**Background:**

Volatile organic compounds such as gasoline and other fuels are associated with a wide variety of deleterious health effects including liver and kidney diseases. Gasoline station workers are exposed to a mixture of hydrocarbons during dispensing. However, no published studies investigated the liver and renal function tests of gasoline station workers in Ethiopia. Therefore the aim of this study was to assess liver and renal function tests among gasoline station workers.

**Method:**

A comparative cross sectional study was conduct from January 2018 to April 2018 at Mekelle city, Tigray region, Northern Ethiopia. Liver and renal function tests were analyzed on gasoline exposed (n = 43) and controls (n = 47) by Pentra C400 automated clinical chemistry analyzer. Student independent t-test and one way-ANOVA statistical methods were employed using SPSS Ver23. P-value < 0.05 was regarded as statistically significant.

**Result:**

The mean level of ALT, AST, Urea, creatinine, and uric acid was significantly higher among gasoline stations workers when compared to control study participants. There was also a significant increase in ALT, AST, Urea, creatinine and uric acid among gasoline stations with above 6 years exposure when compared with those exposed for ≤2 and3-6years.

**Conclusion:**

These findings suggest that increasing liver and renal parameters may be associated with exposure to gasoline and it is dependent on time of exposure to gasoline.

## 1. Introduction

Liver and kidney play a critical role in the body. For example, the liver performs many functions include; metabolism, digestion, detoxification, and stores carbohydrates [1]. The kidneys maintain the blood volume, regulate the mineral content in the bloodstream, and eliminate metabolic wastes from the body [2]. Liver Function Tests (LFTs) and Renal Function Tests (RFT) are the most commonly requested tests for investigation of liver [3] and Kidney disease [4] respectively. The liver function tests include: aspartate aminotransferase (AST), alanine aminotransferase (ALT), alkaline phosphatase (ALP), protein, and bilirubin [5]. Creatinine, urea, uric acid and electrolytes are makers for routine analysis [4].

Elevated levels of LFTs and RFTs may indicate liver [5–7] and renal [4] dysfunction. The elimination of toxic substances is just one of the many functions of the liver and kidneys. Frequent and heavy exposure to gasoline constituents may associate with increased risk of liver [8] and renal [9] function impairment. Gasoline is a volatile and inflammable petroleum-derived liquid mixture of hydrocarbons (such as benzene, toluene, and xylene) and additives [10, 11].

Exposure to benzene, toluene, and xylenes are considered to be the most hazardous, predominantly benzene because of its carcinogenic potency [12, 13]. The health impact of the exposed subjects are due to free radicals, reactive oxygen species (ROS) and reactive nitrogen species (RNS) [14]. The major routes of exposure pathways of gasoline are inhalation, ingestion, and skin contact in which inhalation has more absorption than others [15].

Benzene is metabolized in the liver by cytochrome P450 (CYP) 2E1 to benzene oxide. The metabolites undergo further metabolism through oxidation, dehydrogenation or conjugation with sulfate or glucuronic acid [16, 17]. “Activation of benzene and its reactive metabolites leads to continuous production of reactive oxygen species (ROS), which leads to lipid peroxidation and damages DNA, RNA, leading to genetic modification and alterations in the functions of important enzymes (i.e. liver) and proteins” [18].

Gasoline stations are likely to be an important point source of carbonyl compounds and volatile organic compounds (VOCs), especially emission of benzene, toluene, ethylbenzen, and xylenes (BTEX) [19]. Volatile organic compounds such as gasoline and other fuels are associated with a wide variety of deleterious health effects including liver and kidney diseases [20, 21].

Gasoline station workers are exposed to a mixture of hydrocarbons during dispensing fuel and to the gases from vehicular exhaust [22] and are more risky due to their occupational exposure. Cytotoxic effects of petroleum products are exerted on most of body organs of humans and animals such as the liver and kidney [23]. Exposure to benzene for a long period of time could cause nephrotoxicity and hepatotoxicity and the seriousness of poisoning caused by benzene depends on the amount, rout, and length of time of exposure, as well as the age and preexisting medical condition of the exposed person [24].

Even though, several studies pointed to the risk of occupational exposure to gasoline on liver and renal function profiles, to the best of author’s knowledge no published studies have investigated liver and renal function tests profile of gasoline station workers in Ethiopia. Therefore, the aim of this study was to assess the effect of gasoline exposure on liver (ALT, AST, ALP, total bilirubin) and renal (urea, creatinine, and uric acid) function tests in gasoline station workers.

## 2. Materials and methods

### Study area, design and period

A comparative cross sectional study was conducted from January 2018 to April 2018 at Mekelle City gasoline station workers. Mekelle is capital City of Tigray Regional State and is located in the Northern part of Ethiopia, at 783 km from the capital City of Ethiopia, Addis Ababa.

Mekelle has weyna-dega climatic conditions with an elevation of 2,254 meters above sea level. According to projected Central Statistical Agency of Ethiopia (2017), total population of 358,529 resides in the town. Out of those 50.6% (181,529) of total population are male and 49.4% (176, 98) of total population are female [25]. Administratively Mekelle is divided into 7 sub-Cities; namely: Ayder, Hawelti, Adi Haqi, Hadnet, Kedamay Weyane, Quiha, and Semien.

### Subjects

A total of 43 volunteer gasoline station workers from nine different gas stations in Mekelle city who exposed to gasoline for at least eight hours daily over a period of six months or more, adult male and female aged (18–60) years were participated. On the other hand, the control groups were 47 apparently health workers of Mekelle University Ayder Comprehensive Specialized Hospital. These participants were matched by sex and age with exposed group, and not occupational exposed to gasoline. After explaining the purpose and procedure of the study to all participants, their informed consent was obtained.

### Eligible criteria

#### Inclusion criteria

- Apparently health workers who exposed for gasoline at least 8 hours daily over six months or more in the gasoline station
- Workers who voluntarily participated in the study and sign for consent.

#### Control group

- Workers of Ayder Comprehensive Specialized Hospital who were comparable with gasoline station attendant except for risk of exposure to gasoline.

#### Exclusion criteria

The exclusion criteria of both groups were:

➢ history of hepatic and renal health problem
➢ history of taking medications affecting liver and renal function tests

### Study location

#### Study group (exposed group)

There were a total of 13 legally registered gasoline stations in Mekelle city, but 9 of the 13 were giving fueling service during this study. The study included 43 out of the 49 gas station attendants recruited from 9 gas stations and who volunteered to participate in the study. The gas station attendants were interviewed and a blood sample was collected at administrative office of each station during their workload relatively decreased.

#### Comparison group (control group)

The study included 47 apparently health workers of Ayder Comprehensive Specialized Hospital. The participants were interviewed and a blood sample was collected at central laboratory of Ayder comprehensive specialized hospital during work day.

The sample collected from gasoline exposed and non-exposed participants were analyzed at Ayder Comprehensive Specialized Hospital central laboratory. The demographic detail of study participants were described in Table 1.

**Table 1:** Demographic distribution of study participants.

Number	Exposed group(n = 43)		Comparison group(n = 47)	
	Location(name of gas station)	Number	Location of participants	Number
1	Quiha	2	Clinical staff	23
2	Yetebaberut	6	Academic staff	24
3	Ajib	17		
4	Selam	1		
5	Total oil	12		
6	Oil libya	5		
Total		43		47

### Sample size determination and sampling procedure

Because of few gasoline stations in Mekelle city, all volunteer gasoline station attendants were participated A total of 43 out of 49 gasoline fueling workers and 47 apparently health participants from Ayder Comprehensive Specialized Hospital were included in this study by using convenience sampling method.

### Data collection procedure

Data on age, sex, and employment duration of both groups were collected by administered questionnaire.

### Specimen collection and processing

About 4 ml venous blood sample was collected in to serum separator tube by certified medical laboratory personnel from both groups. The samples of gasoline station workers were collected at administrative office of each gasoline station on routine work hours. Whereas, the control groups sample was collected at Ayder comprehensive specialized hospital central laboratory. Sample was allowed to clot naturally for minimum of 30 minute and serum was separated by centrifuging at 4000 revolution per minute(RPM) for 5 minute.

### Laboratory analysis

Liver function tests (ALT, AST. ALP and total bilirubin) and Renal function tests (urea, creatinine, and uric acid) were analyzed in the serum. The measurements of these parameters were carried out by spectrophotometric determination of their absorbance’s using analytical grade laboratory reagent kits from HORIBA ABX SAS (Parc Euromedecen-Rue du caducee, France) and by Pentra C400 automated clinical chemistry analyzer. All biochemical analysis for this study was according to manufactures protocol.

### Data quality assurance

The questioner was translated to local language Tigrigna to make certain that the participants can understand the questions at the time of interview. Before the actual data collection, questionnaire was pre tested on 5 participants of two gas stations and those participants were excluded from study.

Standard operating procedure was followed during sample collection, transportation, processing, and analyzing. The participants sample was analyzed after both normal and pathologic controls were done and accepted. All data was recorded with identification of each participant, checked for completeness, and interpreted.

### Data analysis and interpretation

Data was analyzed using Statistical Package for Social Science (SPSS) Version 23. To conduct analysis, test of normality was done through Kolmogorov-Smirnov test. Quantitative variables were expressed as mean ± standard deviation, where independent t-test was used to compare between the means of gasoline station workers and control group. One way ANOVA (Analysis of variance) was used to determine duration exposure category of exposed group with liver and renal function tests. P value < 0.05 was considered statistically significant.

### Ethical consideration

The study was conducted after ethically reviewed and approved by the Department of Medical Laboratory Science research and ethical review committee (DRERC), College of Health Science, Addis Ababa University. Permission was obtained from administration of Mekelle cit, each gasoline station, and Ayder Comprehensive Specialized Hospital. Written informed consent to voluntarily participate was obtained from all study participants after explaining the purpose of the study. The confidentiality of the data was also assured.

## 3. Result

### 3.1 Socio demographic characteristics of study participants

A total of 90 study participants comprising of 43 gasoline station workers (28 male and15 female) and 47 controls (30 male and 17female) were recruited for the study. There was no statistically significant age and sex differences between the exposed group and control groups (mean age±SD = 30.02±8.62 vs. 29.85±7.29; P = 0.444) and (sex: male = 28 vs.30, female = 15 vs.17; P = 0.54). All study participants were nonsmoker. Mean of work duration in years and work hour per day of gas station workers was 5.187± 4.39 years (ranged 10 month -16 years) of 11.16±2.08 hours (range 8–14 hours) respectively.

### 3.2 Comparison of liver and renal parameters among gasoline exposed and controls

Study participants who were exposed to gasoline experienced significantly increased mean ALT, AST, urea, creatinine, and uric acid (mg/dL) compared with the unexposed study participants. While mean of ALP (U/L) was significantly lower among gasoline station workers (73.89±16.71 versus 82.91±16.67, p = 0.012) Table 2.

**Table 2:** Comparison of liver and renal function tests among exposed and control study participants in Mekelle City, Tigray region, Northern Ethiopia, January to April, 2018 (n = 90).

Parameter	Exposed group (n = 43)	Comparison group (n = 47)	*p-value
	Mean ±SD	Mean ±SD	
ALT U/L	24.4±10.2	19.06±5.96	0.003
AST U/L	26.26±9.59	20.05±4.55	<0.001
ALP U/L	73.94±16.71	82.91±16.67	0.012
Total B md/dL	0.558±0.289	0.497±0.205	0.251
Urea md/dL	29.82±7.56	20.03±3.69	<0.001
Creatinine mg/dL	0.91±0.14	0.8±0.104	<0.001
UA mg/dL	5.6±1.6	4.24±0.78	<0.001

SD = Standard deviation, U/L = international unit/liter, mg/dL = mill gram/ deciliter

* = independent t-test.

### 3.3 Effect of work duration on liver and renal function tests of gas station workers

The ALT, AST, Urea, uric acid, and Creatinine level of gas station study participants in above six years period of exposure to the gasoline showed significant increase compared with study participants exposed for less than six years Table 3.

**Table 3:** The effect of duration of exposure on liver and renal function tests of gas station workers in Mekelle city, Tigray region, Northern Ethiopia, January to April, 2018 (n = 43).

Test parameter	Duration of Exposure in years		
	≤ 2 years	3–6 years	>6 years
	(mean± SD)	(mean± SD)	(mean± SD)
ALT (U/L)	17.5±5.9	22.4±4.8	34.5±1.1^**a,b**^
AST (U/L)	20.9±5.3	24.3±4.8	34.4±11.5^**a,d**^
ALP (U/L)	70.9±22.4	73±10.1	78.3±12.96
TB (mg/dL)	0.53±0.33	0.50±0.25	0.64±0.28
Urea (mg/dL)	26.5±6.4	26.7±5.3	36.5±6.3^**a,b**^
Creatinine (mg/dL)	0.85±0.1	0.87±0.9	1.03±0.1^**a,d**^
Uric acid (mg/dL)	5.09±1.6	5.16±1.3	6.6±1.4^**c,d**^
Total	17	12	14

• F-test (One way-Anova) with post hoc multi comparison is used to compare means.

➢ a-p<0.001 compared to ≤ 2 years (One-way ANOVA/tukey post hoc test).

➢ b- p<0.001 compared to 3–6 years (One-way ANOVA/tukey post hoc test).

➢ c- p<0.05 compared to ≤2 years (One-way ANOVA/tukey post hoc test).

➢ d- p<0.05 compared to 3–6 years (One-way ANOVA/tukey post hoc test).

## 4. Discussion

Gasoline station workers are regularly exposed to many hazardous toxins vapors of gasoline, kerosene, and diesel, among the most risky toxin is benzene fume. Which can cause abnormal alterations in the functioning of many vital organs and they are associated with increased risk of renal and liver cancer [26].

The findings of the present study indicate that gasoline exposure can induce significant alterations in renal and hepatic functions among gasoline exposed study participants. Specifically; ALT, AST, Urea, creatinine, and uric acid are significantly higher among gasoline stations workers when compared to control study participants. ALP level was significantly lower among gasoline exposed subjects compared to unexposed study participants. However, Total bilirubin level was statistically no significant between exposed and unexposed study participants.

The significant increase in the creatinine and urea levels of the gasoline station workers compared with the control in our study was in agreement with the findings in previous studies conducted in Nigeria, Iraq, Palestine, and Saudi Arabia [20, 21, 26–28] who gasoline station attendants to have altered renal function values. This may be attributed to an increase in liberating toxic metabolites as reactive oxygen species (ROS). Some experiments with rats indicate that exposure by inhalation to aromatic hydrocarbons can cause nephrotoxic. Furthermore human and experimental studies suggest that some of chemicals can affect the renal system. Since petroleum products (benzene) are chemicals it can be a cause of the renal impairment [22]. Serum uric acid level was also significantly increase which is in line with studies done in Iraq and Palestine [26, 29]. This may be due to degradation of purines or an increase of uric acid level by either over production or inability of excretion [26].

Serum ALT and AST were significantly higher in gasoline station workers than controls study participants while ALP was significantly decreased in exposed study participants than controls. Whereas, Total bilirubin had no significantly different between exposed and controls. The significantly increase in ALT and AST is in line with other studies conducted in Egypt, Nigeria, Turkey, Palestine, India, and Brazil [13, 30–34]. This observation may be due to the fact that hydrocarbons which are a major component of petroleum products that are metabolized in the liver by CYP450 2E1 oxidative pathways which contribute to the production of free radicals and quinine metabolites such as phenol, hydroquinone, benzoquinone;1,2,4 benzenetriol [17]. These free radicals and toxic metabolites cause lipid peroxidation and damage of hepatic cell membrane, causing the release of liver enzymes in the circulation [35].

The ALP level significantly lower in exposed subjects than controls is in line with study conducted in Nigeria by Akinosun O. M et al [36]. ALP is an enzyme mostly found in the cells lining the biliary ducts of the liver. If there is an obstruction in the bile duct, ALP may leak into blood stream and ALP levels in plasma will rise [37]. ALP is also present in placental tissue and in growing children for bone remodeling [26]. All the study participants considered for this study are adult; therefore, significantly low levels of ALP in petrol station attendants indicate absence of bile duct obstruction. Similar levels of total bilirubin in petrol attendants and controls are an indication that no hemolysis or liver damage occurred in the petrol attendants.

In our study the effect of time exposure to petroleum products and their derivatives on liver and renal parameters of gasoline station workers was assessed. Liver function tests such as; ALT, AST level of gas station study participants in above 6 years period of exposure to the gasoline showed significant increase compared with participants exposed for less than or equal to two years and 3–6 years of work duration. Previous reporters on human and animals demonstrated that many chemicals can affect the kidney and liver function. These finding are in agreement with the earlier report in Egypt, Nigeria, and Iraq [21, 27, 28, 38]. Renal function test (urea and creatinine) level of study participants with above 6 years of work duration was also significantly higher compared with study participants within less than or equal to two years and 3–6 years of work duration. Other reports confirmed that petrol products may have some effects on kidney functions [21, 23, 26, 28].

## 5. Conclusion

The exposure to petroleum products at gasoline stations workers showed that an increase of liver and renal parameters. This study observed that the gasoline station workers are at risk of developing biochemical alterations in the hepatic enzymes and renal function tests. The liver and renal parameters were significantly increased with duration of exposure of gasoline station attendants. That indicates increase the probability of liver and kidney function tests among gasoline stations workers with increased exposure time.

## Supporting information

S1 File (DOCX)

S2 File (DOCX)

S1 Data (XLSX)
