# Supplementary material for: Assessment of liver and renal function tests among gasoline exposed gas station workers in Mekelle city, Tigray region, Northern Ethiopia
Source: PLoS One. 2020 Oct 9;15(10):e0239716. doi: 10.1371/journal.pone.0239716 (PMC7546501; doi:10.1371/journal.pone.0239716)
Supplement: S1 File — (DOCX) [file pone.0239716.s001.docx]

## **Structured Questionnaire for Gasoline exposed Study participants**

Assessment of liver and renal function tests among gasoline exposed gas station workers in Mekelle city, Tigray region, North Ethiopia

### A. English Version

**Identification:**

Name of facility (optional)_____________ Institution code_______________

Kifle Ketema__________ Woreda__________ Kebelle_________ Tel: -----------------

Respondent’s identification code: ___________

**Note: please encircle or write the appropriate answer on the space provided.**

**I. Socio-demographic characteristics**

1. Sex: 1. Male 2. Female

2. Age (Years): ______________________________

**II. Health related associated factors**

3. How long have you worked in the gas station? (Years of work)..........

4. How many hours you work at this station per day……….

5. Do you smoke tobacco/cigarette? (1) Yes (2) No

6. If your answer to question No 12 is Yes, How many pieces of cigarettes on average do you smoke per day?

7. Do you have a history of any liver or kidney problem before hiring to this work?

(1) Yes (2) No

**I thank you for your cooperation!**

### B. Amharic Version

**መለያ:** የተቋሙ ስም _________________ የተቋሙ ኮድ:-----------------የተሳታፊዎችመለያቁጥር_______________

ክፍለ ከተማ__________ ወረዳ__________ ቀበሌ_________ ስልክ: -----------------

**የጥናቱ ርእስ:** “በነዳጅ ማድያና ድርጅት የሚሰሩና በስራቸው ምክንያት በነዳጅ የሚጠቁ ሰራተኞች በደማቸው ውስጥ ያለውን የጉበትና የኩላሊት ጤንነት ጠቋሚም ርመራዎች መጠን መለካት”

**ማሳሰብያ**: እባክዎ ትክክለኛዉን የሆነዉን መልስ ያክብቡ ወይም ይፃፉ፡፡

**መጠይቅ አንድ: ማህበራዊ ተጨባጭ ሁኔታዎች**

1. ፆታ      (1) ወንድ             (2) ሴት

2. ዕድሜ­­­­­­­­­­­­­­­­­­­­­­­­--------------

**መጠይቅ ሁለት: ከጤና ጋረ ተያያዥነገሮች**

3. በዚህ ስራ ውስጥ ለስንት ግዜ ያህል ሰርተዋል?--------------------------

4. በስራ ምድብዎ በቀን ስንት ሰዓት ይሰራሉ?____________

5.ስጋራ ይስባሉ (ያጨሳሉ)? (1) ኣዎ   (2) አላጨስም

6.ለጥያቄ ቁጥር12መልስዎ አዎ ከሆነ, በቀን በአማካኝ ስንት ፓክ(pack)ስጋራ ይጠቀማሉ?

7. እዚህ ስራ ከመጀመርዎ በፊት የኩላሊት ወይም የጉበት በሽታ ተይዞው ያውቃሉ?

(1) ኣዎ   (2) አላውቅም

**ስለትብብርዎ እናመሰግናለን!**

### C. Tigrigna Version

መፍለዪ**:** ናይቲ ትካል ሽም:_________________ናይቲ ትካል ኮድ:___________ናይ ተሳተፍቲመፍለዪቁፅሪ

ክፍለ ከተማ__________ ወረዳ__________ቀበሌ_________ ስልኪ: -----------------

**ናይቲ መፅናዕቲ ኣርእስቲ**: አብነዳዲመዐደሊ ዝሰርሑን ብስርሖም ምኽንያት ብነዳዲ ዝጥቅዑ ሰራሕተኛታት አብደሞም ውሽጢ ዘሎ ናይ ፀላም ከብዲ(ጉበት) ንኩላልትን ጥዕና ጠቆምቲ ምርመራታት መጠን ምዕቃን

**መተሓሳሰቢ:** በጃኹም ትኽክለኛ ዝኾነ መልሲ የክብቡ ወይ ከዓ ይፅሓፉ።

**1ይ ክፋል: ናይ ማሕበራዊን ነባራዊን ኹነታት ዝምልከቱ ሕቶታት**

1. ፆታ      (1) ተባዕታይ             (2) አንስታይ

2. ዕድመ­­­­­­­­­­­­­­­­­­­­­­­­___________________

**2ይ ክፋል**: **ምስ ኩነታት ጥዕና ዝተተሓሐዙ ነገራት**

3. አብዚ ስራሕ ውሽጢ ክንደይ ግዜ ዝአክል ሰርሖም/ሐን?________________________

4. አብ ስራሕ ምድቦም አብ መዓልቲ ክንደይ ሰዓት ይሰርሑ/ሓ? ______________________________________________

5.ሽጋራ ይስሕቡ/ባዶ(የጭሱ/ሳ ዶ)? (1) እወ   (2) አያጨስን

6.ንሕቶ ቑፅሪር 12 መልሶም/ሰን እወ እንተኾይኑ, ብመዓልቲ ብማአኸላይ ክንደይ ፓክ(pack) ሽጋራ ይጥቀሙ/ማ?

7. አብዚ ስራሕ ቕድሚ ምጅማሮም/ረን ናይ ኩላሊት ወይከዓ ጉበት(ፀላም ከብዲ) ሕማም ተታሕዞም ይፈልጡ/ጣ ዶ?

(1) እወ   (2) አይፈልጥን

19. ቕድሚ ሐዚ ወይከዓ ሐዚ ዝወስድዎ ናይ መድሓኒት ዓይነት እንተልዩ ይፅሓፉ/ፋ----------------------

**ስለዝተሓባበሩና ነመስግን!**
