# Supplementary material for: Assessment of liver and renal function tests among gasoline exposed gas station workers in Mekelle city, Tigray region, Northern Ethiopia
Source: PLoS One. 2020 Oct 9;15(10):e0239716. doi: 10.1371/journal.pone.0239716 (PMC7546501; doi:10.1371/journal.pone.0239716)
Supplement: S2 File — (DOCX) [file pone.0239716.s002.docx]

Code: ____________________

## Annex II: Consent form

### A. English version

**Principal investigator**: Tsegay Asefaw Kahsay

**Advisors:** Mistire Wolde (MSc, PhD)

**Funded by**: Mekelle University

**Reviewed**: By department research ethics committee of department of Medical Laboratory Science, Addis Ababa University

**Research title**: Assessment of liver and renal function tests among gasoline exposed gas station workers in Mekelle City, Tigray region, North Ethiopia.

If you agree to take part, please read this form and sign the consent sheets at the end. Please tick off every box, if you agree.

1. I have read, or it was read to me, the information sheet concerning this study and I understand what will be required of me if I take part in the study.
2. I am aware of the possible risk and benefits of this study.
3. I know that being in this study is voluntary.
4. I understand that at any time I may withdraw from this study without giving reason and without affecting my normal care.
5. My questions concerning this study have been answered by …………………
6. I know that no special payment for being participating in the study.
7. I agree to take part in this study.

Name of participant.___________ Age____Address___________Signature______Date_______

Interviewer’s name___________________________________Signature___________________

Date of interview___________________Time started_____________ Time finished__________

Principal investigator Name______________________ Signature______________________

**I thank you for your cooperation!**

ኮድ:___________

### B. Amharic Version

**የፍቃደኝነት ማረጋገጫ ሰነድ**

**የአጥኝው ስም:** ፀጋይ አሰፋው ካሕሳይ

**ኣማካሪዎች** 1) ዶ/ር ሚስጥረ ወልዴ

2) ኣበበ ኢዳኦ

**የተቋሙ ስም:** አዲስ አበባ ዩኒቨርሲቲ የህክምና ና ጤና ሳይንስ ኮሌጅ የሕክምና ላቦራቶሪ ትምህርት ክፍል

**ስፖንሰር ያደረገው ድርጅት:** መቐለ ዩኒቨርስቲ

**ፍቃድ ሰጪ:**የህክምና ላቦራቶሪ ትምህርት ክፍል የምርምርና ሥነ-ምግባር ቢሮ

**የጥናቱ ርእስ:**“ በተለያዩ የነዳጅ ማደያ የሚሰሩ ሰራተኞች በቤንዚን ምክንያት በደማቸው በተለያዩ የደም ዓይነቶች ላይ የሚያመጣው ጉዳት መጠኑን ለማወቅ ነው ።

ለመሳተፍ ከተስማሙ እባክዎ ይህን ቅጽ ያንብቡ እና በመጨረሻም የስምምነት ወረቀቶችን ይፈርሙ።

እባክዎ ከተስማሙ እያንዳንዱ ሳጥን ላይ ምልክት ያድርጉ።

1. ይህንን ጥናት በተመለከተ የተጻፈውን መረጃ አንብቤያለሁ። እና በጥናቱ ላይ ከተካፈልኩኝ ምን እንደሚጠበቅብኝ ተረድቻለሁ።
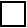


2. በዚህ ጥናት ሊኖር ስለሚችለው አደጋ እና ጥቅሞች አውቂያለሁ።
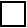


3. በዚህ ጥናት ውስጥ መሳተፌ በፍቃደኝነት መሆኑን አውቂያለሁ ።
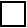


4. በማንኛውም ጊዜ እኔ ምንም ሳልሆን እና መደበኛ እንክብካቤዬን ሳይነካ ከዚህ ጥናት ልወጣ እችላለሁ።
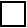


5. በዚህ ጥናት ውስጥ ያሉኝ ጥያቄዎች በመረጃ ሰበሳቢው ተመልሰውልኛል።
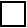


6. በጥናቱ ውስጥ ለመሳተፍ ምንም ልዩ ክፍያ እንደሌለ አውቂያለሁ።
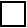


7. በዚህ ጥናት ለመሳተፍ እስማማለሁ።
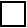


የተሳታፊው ስም ___________________አድራሻ___________ፊርማ ___________ቀን ___________

በስምምነቱን ቅፅ ማንበብ የማይችሉ ተሳታፊዎች

የአማካሪ ስም__________________ አድራሻ___________ፊርማ_________________ቀን__________________

የመረጃ ሰብሳቢ ስም ____________________ፊርማ __________ ቀን__________________

ዋና ተመራማሪ ስም __________________________ ፊርማ___________ቀን_____________

**ስለትብብርዎ እናመሰግናለን!**

ኮድ-----------------

### C. Tigrigna Version

**ናይ ፍቓደኛነት መረጋገፂ ዓንቀፅ**

**ናይ መፅናዓይ ሸም:** ፀጋይ አሰፋው ካሕሳይ

**ኣማከርቲ** 1) ዶ/ር ሚስጥረ ወልዴ

2) ኣበበ ኢዳኦ

**ናይቲ ትካል ሸም:** አዲስ አበባ ዩኒቨርሲቲ ጥዕናን ሕክምናን ሳይንስ ኮሌጅ ናይ ሕክምና ላቦራቶሪ ትምህርቲ ክፍሊ

**ስፖንሰር ዝገበሮ ድርጅት:** መቐለ ዩኒቨርስቲ

**ፍቓድ ወሃቢ:** አዲስ አበባ ዩኒቨርሲቲ ጥዕናን ሕክምናን ሳይንስ ኮሌጅ ናይ ሕክምና ላቦራቶሪ ትምህርቲ ክፍሊናይ ምርምርን ሥነ ምግባርን ቢሮ

**ናይቲ መፅናዕቲ ርእሲ፡-**አብ ነዳዲ መዐደሊዝሰርሑን ብስርሖም ምኽንያት ብነዳዲ (ቤኒዚን) ዝጥቅዑ ሰራሕተኛታት አብ ደሞም ውሽጢ ዘሎ ናይ ፀላም ከብዲ (ጉበት)ን ኩላልትን ጥዕና ጠቆምቲ ምርመራታት መጠን ምዕቃን

አብዚ መፅናዕቲ ንምስታፍ ፍቓደኛ እንተኾይንኹም፤ በጃኹም/ኽን እዚ ቐፅሉ ዘሎ ዓንቀፅ ብምንባብ አብቲ ናይ ስምምዕ ወረቐት ይፈርሙ። ስለዚ አብ ሕድሕድ ሳንዱቕ ምልክት ይግበሩ።

1. ብዛዕባ እዚ መፅናዕቲ ዝተፅሓፈ ናይ ሓበሬታ ወረቐት ኣንቢበዮ ወይከዓ ተነብቡለይ አብዚ መፅናዕቲ ንምስታፍ እንታይ ከምዝድለ ተረድኡኒ እዩ።
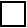


2. አብዚመፅናዕቲ ክህልዩ ዝኽእሉ ፀገማትን ጥቕሞምን ፈልጠ እየ።
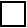


3. አብዚ መፅናዕቲ ንምስታፍ ብፍቐደኛነት ምኻኑ ተረዲኡኒ እዩ።
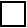


4. ካብዚ መፅናዕቲ አብ ዝኾነ ይኹን ሰዓት ምቁራፅ ከምዝኽእል ተረድኡኒ አሎ።
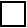


5. አብዚመፅናዕቲ ውሽጢዝነበሩኒ ሕቶታት ተመልስለይ እዩ።
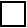


6. አብዚ መፅናዕቲ ውሽጢ ብምስታፈይ ምንም ዓይነት ናይ ገንዘብ ኽፍሊት ከምዘይወሃበኒ ፈሊጠ እየ።
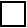


7. አብዚ መፅናዕቲ ንምስታፍ ይስምማዕ አለኹ።
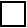


ናይ ተሳታፊ/ፊት ሽም___________________አድራሻ__________ ፊርማ ________ዕለት ___________

አብቲ ናይ ስምምዕ ዓንቀፅ ምንባብ ንዘይኽእሉ ተሳተፍቲ

ናይ አማኻሪ ሽም__________________አድራሻ_________ፊርማ____________ዕለት________________

ሓበሬታ ሰቢሳቢ ሽም ____________________ፊርማ __________ ዕለት__________________

ናይ መፅናዓይ ሽም __________________________ ፊርማ___________ዕለት______________

**ስለዝተሓባበሩና ነመስግን!**
